# On the Possibility of Helium Adsorption in Nitrogen Doped Graphitic Materials

**DOI:** 10.1038/s41598-020-62638-z

**Published:** 2020-04-02

**Authors:** Sudhir K. Sahoo, Julian Heske, Sam Azadi, Zhenzhe Zhang, Nadezda V. Tarakina, Martin Oschatz, Rustam Z. Khaliullin, Markus Antonietti, Thomas D. Kühne

**Affiliations:** 1 Dynamics of Condensed Matter and Center for Sustainable Systems Design, Chair of Theoretical Chemistry, University of Paderborn, Warburger Str. 100, D-33098 Paderborn, Germany; 2 Department of Colloid Chemistry, Max Planck Institute of Colloids and Interfaces, Am Mühlenberg 1, D-14476 Potsdam, Germany; 3 Department of Physics, King’s College London, Strand, London, WC2R 2L United Kingdom; 4 Department of Physics, Imperial College London, Exhibition Road, London, SW7 2AZ United Kingdom; 5 Department of Chemistry, McGill University, 801 Sherbrooke Str. West, Montreal, Quebec H3A 0B8 Canada; 6 University of Potsdam, Institute of Chemistry, Karl-Liebknecht-Str. 24–25, D-14476 Potsdam, Germany; 7 Paderborn Center for Parallel Computing and Institute for Lightweight Design, University of Paderborn, Warburger Str. 100, D-33098 Paderborn, Germany

**Keywords:** Theoretical chemistry, Porous materials, Two-dimensional materials

## Abstract

The potassium salt of polyheptazine imide (K–PHI) is a promising photocatalyst for various chemical reactions. From powder X–ray diffraction data an idealized structural model of K–PHI has been derived. Using atomic coordinates of this model we defined an energetically optimized K–PHI structure, in which the K ions are present in the pore and between the PHI–planes. The distance between the anion framework and K^+^ resembles a frustrated Lewis pair-like structure, which we denote as frustrated Coulomb pair that results in an interesting adsorption environment for otherwise non-adsorbing, non-polar gas molecules. We demonstrate that even helium (He) gas molecules, which are known to have the lowest boiling point and the lowest intermolecular interactions, can be adsorbed in this polarized environment with an adsorption energy of  − 4.6 kJ mol^−1^ per He atom. The interaction between He atoms and K–PHI is partially originating from charge transfer, as disclosed by our energy decomposition analysis based on absolutely localized molecular orbitals. Due to very small charge transfer interactions, He gas adsorption saturates at 8 at%, which however can be subject to further improvement by cation variation.

## Introduction

Materials based on carbon-nitride (CN) have received huge attention in the scientific community for their large variety of applications in catalysis^1–5^, gas storage and separation^6^, battery research^7–9^, and other energy storage devices^10^. Recently, a new CN material, potassium salt of polyheptazine imide (K–PHI), has been synthesized, which, beside promising catalytic activity for various organics^11^ and photocatalytic water–splitting reactions^12–14^, also obeys unexpected sorption properties. As this material has a definite pore size, it is characterized by the presence of extra-framework cations and is thus, due to its hydrophilic nature, potentially relevant for gas storage and separation. In fact, it has been already demonstrated that the hydrogen gas (H_2_), which is non-polar and easily boiling, can be adsorbed over metal-free CN materials^6^. The adsorption of H_2_ gas on Li-decorated N-doped graphene has also been modelled theoretically^15^. In pure organic systems, H_2_ gas is adsorbed and activated usually by frustrated Lewis pairs (FLPs)^16–19^.

In K–PHI, the 2D PHI–layers are negatively charged and have base character, while K^+^ is positively charged and can be, when non-solvated, understood as a form of generalized solid state acid, thus the whole system indeed resembles a FLP-like structure. Therefore, K–PHI could be a suitable candidate for the non-polar gas adsorption, in its extreme, exemplified by helium (He) gas. It is noted that He gas has the lowest boiling point ( ≈  4.3 K) of all known molecules, and liquefying He gas is technologically demanding. Adsorption materials, which are another option for storage and transportation of He gas, share a similar problem: the polarizability of He is so low that there is little He adsorption at all. On the one hand, this weakness of interaction of He with various metals/materials is exploited because of its inertness and low solubility in various materials, reference states can be marked^20–25^. On the other hand, He adsorption materials would therefore enable new technologies and are hence in high demand. Schierholz *et al*.^26^ have reported that using magnetron sputtering to generate nanoporous amorphous silicon, high storage of He gas (21 at%) can be achieved. With a similar method,  ≈  16 at% He was observed in titanium alloy films^27^. The authors also reported that the He atoms are distributed homogeneously throughout the films, while small bubbles are formed. However, to best of our knowledge, there has been neither experimental nor theoretical work on He gas adsorption in CN-based materials.

In this paper, we investigate whether K–PHI could be used for He gas adsorption and storage by employing density functional theory (DFT) and quantum Monte Carlo calculations. Yet, it is important to mention that the K ions in the K–PHI lattice can be exchanged with other ions^14^. This allows to tune the nature of the material, which might also affect its surface properties. Therefore, to elucidate the effect of ions within the PHI–scaffold on the He adsorption we have chosen H–PHI as our reference system. Moreover, Au–PHI was elected to investigate the impact of different cations on the structure and hence He adsorption.

## Results

Our reference structure H–PHI was synthesized from K–PHI in an ion-exchange process, as described in ref. ^14^. From the powder X–ray diffraction pattern, the structure of H–PHI can be indexed in hexagonal lattice with the unit cell parameters *a* = *b* =  12.5 Å and *c* =  3.2998 Å, respectively. All of our DFT calculations were carried out with these lattice parameters. Based on our calculations, we find that the bridging N atoms are covalently bonded to the hydrogen atoms, as shown in Supplementary Fig. S1. This goes well with the fact that H–PHI is a weak acid.

**Figure 1:**
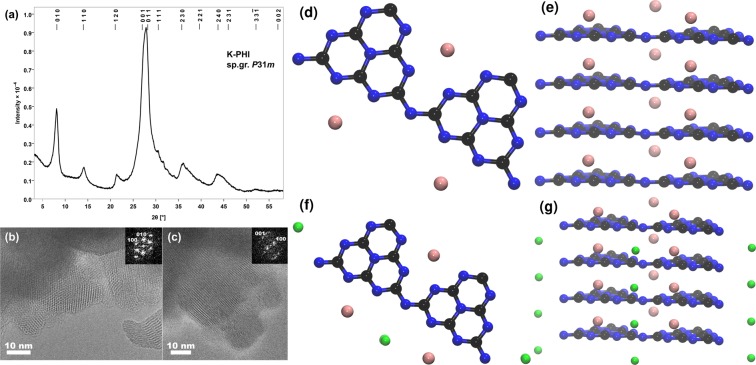
The powder X–ray diffraction pattern of K–PHI indexed in space group *P*31*m* (**a**) and HRTEM images of K–PHI (**b–c**) with Fast Fourier transforms in the insets. The atomic structure of bulk K–PHI, where all K ions are present between the PHI–layers (**d**–top view and **e**–side view). The atomic structure of He@K–PHI (**f**–top view and **g**–side view), where twelve He atoms are adsorbed in K–PHI. All He atoms are present between the PHI–planes. The C, N, K and He atoms are shown in black, blue, pink and green, respectively.

In comparison to H–PHI, the atomic structures of K–PHI and Au–PHI are more complicated due to the presence of extra-framework cations. Yet, images from high-resolution transmission electron microscopy (HRTEM), which are shown in Fig. 1, shows the crystalline nature of K–PHI. Again, from the PXRD pattern the structure of K–PHI was found to be on a hexagonal lattice, though with slightly expanded lattice parameters *a* = *b* =  12.637 Å and *c* =  3.2998(3) Å, respectively. Both H–PHI and K–PHI structures can be described as a stacking of heptazine units with continuous channels formed along the *c* direction. But, due to the high concentration of stacking defects and turbo-static disorder in the crystals, the positions of the K ions within the K–PHI lattice could not be determined. Hence, in order to localize the K ions in the K–PHI lattice, the protons within H–PHI were replaced in the model by K ions, followed by a dynamical simulated-annealing^28,29^ using the second-generation Car-Parrinello molecular dynamics approach by Kühne *et al*.^30,31^ to locate the nuclear ground-state (see Supplementary Fig. S2).

From these calculations, we conclude that the K ions are located between the PHI–layers in the pores of K–PHI, as can be seen in Fig. 1. Using the so calculated geometry of K–PHI as input to refine the description of the PXRD pattern and to estimate the eventual distribution of the K ions, we obtain an atomic structure that qualitatively agrees well with the theoretical model (see Supplementary Fig. S3). The structure of Au–PHI was also predicted in similar way, leading to Au atoms that are located in the pores, but in the same plane as the 2D PHI scaffold (see Supplementary Fig. S4).

For the purpose to quantify possible He adsorption in K–PHI, H–PHI and Au–PHI, we compute the total adsorption energy $\Delta {E}_{{\rm{tot}}}^{{\rm{ads}}}$ per He atom as

1 $$\Delta {E}_{{\rm{tot}}}^{{\rm{ads}}}=\frac{1}{n}\{E(n{\rm{He}}@{\rm{M}}-{\rm{PHI}})-E({\rm{M}}-{\rm{PHI}})-nE({\rm{He}})\},$$

as well as the incremental adsorption energy

2 $$\Delta {E}_{{\rm{incr}}}^{{\rm{ads}}}=E(n{\rm{He}}@{\rm{M}}-{\rm{PHI}})-E((n-1){\rm{He}}@{\rm{M}}-{\rm{PHI}})-E({\rm{He}}),$$

where *E*(*n*He*@*M − PHI) is the potential energy of the system when *n* He atoms are adsorbed in M–PHI, whereas *E*(M - PHI) and *E*(He) are the potential energies of M–PHI and an individual He atom, respectively. A negative value for the adsorption energy indicates that the He adsorption is thermodynamically favourable. The corresponding results of $\Delta {E}_{{\rm{tot}}}^{{\rm{ads}}}$ and $\Delta {E}_{{\rm{incr}}}^{{\rm{ads}}}$ for the He adsorption in K–PHI are shown in Table 1. Interestingly, the He atoms can occupy both sites, either in the same plane as the 2D PHI scaffold or between them, as can be seen in Supplementary Fig. S5 and Fig. 1. Nevertheless, we find that in case of the between the PHI–plane positions, the total adsorption energy is linearly increasing with the number of adsorbed He atoms and as such thermodynamically much more favorable. Similarly, $\Delta {E}_{{\rm{tot}}}^{{\rm{ads}}}$ and $\Delta {E}_{{\rm{incr}}}^{{\rm{ads}}}$ for H–PHI (Supplementary Table S1) and Au–PHI (Supplementary Table S2) are indicating that He adsorption is thermodynamically favourable for these systems as well. The corresponding structures are shown in Supplementary Figs. S6–S8.

**Table 1:** Adsorption energy in kJ mol^−1^ for He adsorption in K–PHI.

No. of He atoms	He atoms in the same PHI–plane		He atoms between the PHI–plane	
	${\boldsymbol{\Delta }}{{\boldsymbol{E}}}_{{\bf{tot}}}^{{\bf{ads}}}$	${\boldsymbol{\Delta }}{{\boldsymbol{E}}}_{{\bf{incr}}}^{{\bf{ads}}}$	${\boldsymbol{\Delta }}{{\boldsymbol{E}}}_{{\bf{tot}}}^{{\bf{ads}}}$	${\boldsymbol{\Delta }}{{\boldsymbol{E}}}_{{\bf{incr}}}^{{\bf{ads}}}$
1	− 4.5	− 4.5	− 4.2	− 4.2
2	− 4.7	− 4.8	− 4.3	− 4.3
3	− 4.5	− 4.2	− 4.2	− 4.2
4	− 4.7	− 5.2	− 4.3	− 4.6
5	− 4.2	− 2.3	− 4.2	− 3.5
6	− 4.2	− 4.2	− 4.1	− 3.8
7	− 3.9	− 2.1	− 4.1	− 3.8
8	− 4.2	− 5.7	− 4.0	− 3.7
9	− 3.7	0.03	− 4.0	− 3.5
10	− 3.2	1.0	− 3.9	− 3.0
11	− 2.8	0.8	− 3.9	− 3.6
12	− 2.5	0.8	− 3.8	− 3.5

It is important to highlight that $\Delta {E}_{{\rm{t}}{\rm{o}}{\rm{t}}}^{{\rm{a}}{\rm{d}}{\rm{s}}}$ for K–PHI is around 1 kcal mol^−1^, which is within the accuracy limit of semi-local DFT calculations^32^. Hence, in order to validate our DFT results, we carried out rather accurate many-body wave function based reference calculations using the quantum Monte Carlo method^33–35^. Specifically, first a variational Monte Carlo simulation was conducted to yield a many-body trial wave function^36,37^, which is then used as a guiding wave function for an even more accurate fixed-node diffusion Monte Carlo (DMC) calculation^38,39^. The exact computational details are described in the supplementary information. The eventual total DMC adsorption energy for six adsorbed He atoms within K–PHI equals to  − 4.6(1) kJ mol^−1^. This is to say that the employed dispersion-corrected semi-local DFT calculations are in qualitative agreement with accurate many-body wave function based theories, but also that our primary calculations yield a lower bound that underestimates the true adsorption energy by  ≈ 0.5 kJ mol^−1^.

**Figure 2:**
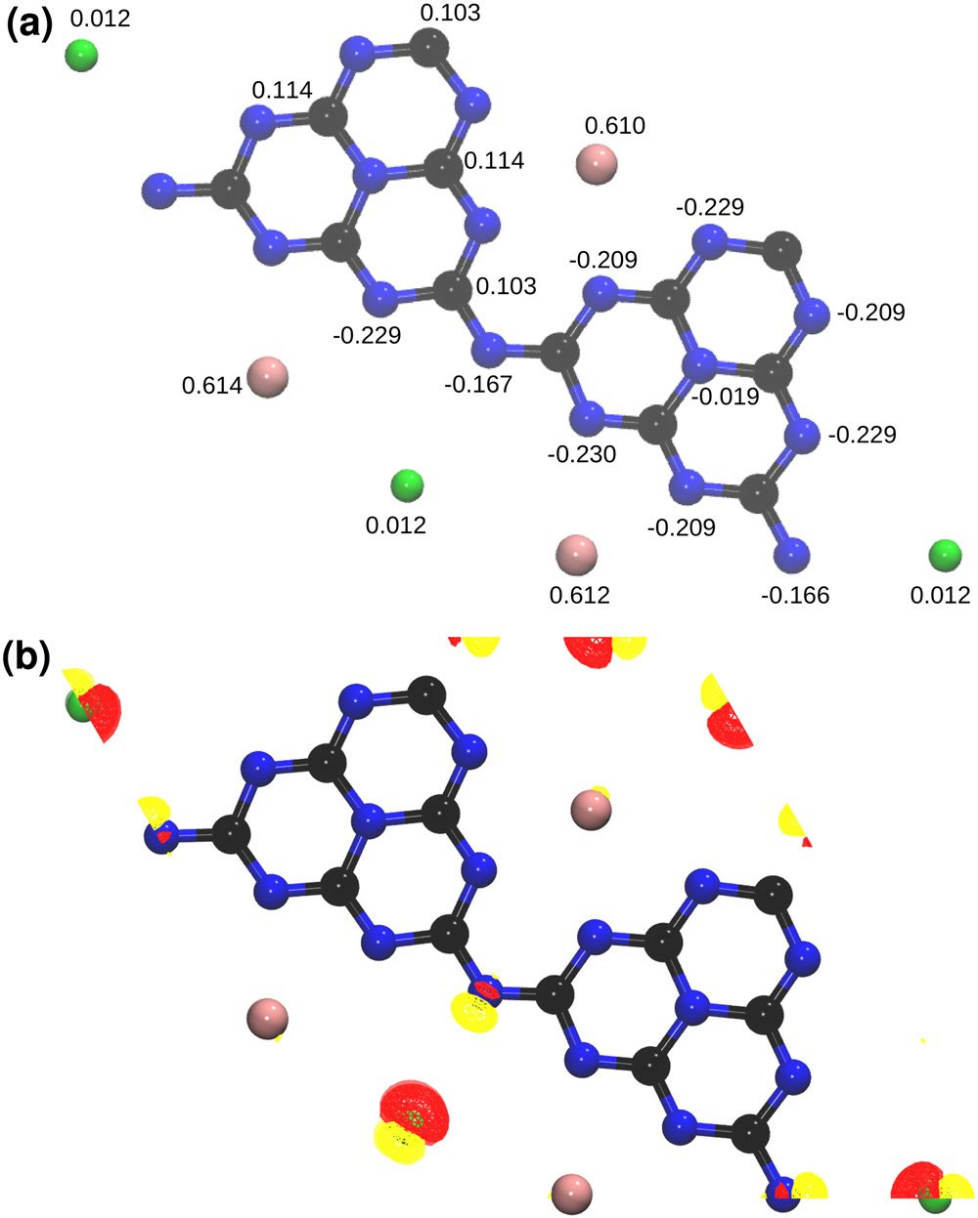
Mulliken populations analysis of He@K–PHI where all the He atoms are present between PHI–layers (**a**). Electron density difference plot (isovalue =  ± 0.0003) of He@K–PHI (**b**). Red and yellow colors represent the depletion and accumulation of electron density, respectively.

To provide an illustrative means of estimating the partial atomic charges, we have performed a Mulliken population analysis of K–PHI^40^, which of course reflects that the K ions have a positive charge, whereas the PHI–layer is negatively charged, as shown in Supplementary Fig. S9. Identically obtained partial atomic charges for H–PHI and Au–PHI are displayed as Supplementary Figs. S10 and S11, respectively. More excitingly, Fig. 2a demonstrates that from the otherwise inert He a partial charge amounting to 1.2% of an electron is moved to the rather electron-poor K–PHI scaffold, i.e. the He binds to the PHI framework by partial charge transfer. More sophisticated Density Derived Electrostatic and Chemical (DDEC) net atomic charges^41^, which are chemically more meaningful and reproduces the electrostatic potential accurately, confirms our findings of the Mulliken population analysis. The corresponding DDEC net atomic charges are shown in Supplementary Figs. S12–S14.

In addition to the comparably large partial charge transfer, a second contribution to binding comes from the polarization of the He atoms creating a dipole moment in the electron distribution around the nucleus as a reaction to the outer frustrated Coulomb field in the PHI. For better visualization, we have computed the electron density difference

3 $$\Delta \rho =\rho ({\rm{H}}{\rm{e}}@{\rm{M}}-{\rm{P}}{\rm{H}}{\rm{I}})-\rho ({\rm{M}}-{\rm{P}}{\rm{H}}{\rm{I}})-\rho ({\rm{H}}{\rm{e}}),$$

where *ρ*(H*e@M* − *PHI*) is the total electron density of He@M–PHI, while *ρ*(M − PHI) and *ρ*(He) are the total electron densities of M–PHI and the He atoms. From Fig. 2b, where the difference in electron density due to the interactions are colour-coded, it is apparent that at all He positions roughly the same polarized electron distribution is induced. We hence deduce that the FLP-like structure of K–PHI, which we therefore denote as frustrated Coulomb pair (FCP) from now on, is able to polarize He between the charge centers, thereby contributing to the He adsorption being thermodynamically favourable. Assuming that charge transfer effects due to the FCP structure of K–PHI are essential for the adsorption of He, this would also explain, why the adsorption energies for K–PHI a systematically higher than for Au–PHI and H–PHI, respectively, where the Au and H atoms are located in the same plane as the 2D PHI scaffold and create less frustration.

In order to rigorously quantify the nature of interactions between He atoms and the M–PHI scaffold, the absolutely localized molecular orbital based energy decomposition analysis (ALMO–EDA), developed by some of us for periodic systems^42–45^, was conducted. Using ALMO–EDA, the total interaction energy between the He atoms and the M–PHI scaffold is decomposed into chemically meaningful components, as shown in Table 2.

**Table 2:** The total interaction energy *ΔE*_TOT_ between the He atoms and the M–PHI scaffold is decomposed into physical meaningful components, i.e. frozen energy *ΔE*_FRZ_, polarization energy *ΔE*_POL_ and charge transfer energy *ΔE*_CT_, using the ALMO–EDA technique. All energies are given in kJ mol^−1^.

	*ΔE*_FRZ_	*ΔE*_POL_	*ΔE*_CT_
He@H–PHI	− 23.74	− 0.599	− 11.37
He@Au–PHI	− 0.45	− 4.52	− 14.98
He@K–PHI	− 31.03	− 3.28	− 11.93

Our calculations suggest that the charge transfer term is sizably contributing to the total interaction energy, though we found that the stabilization energy due to charge transfer is rather similar in all three systems investigated here and only slightly higher in the case of He@Au–PHI. In the case of He@H–PHI and He@K–PHI, the maximum contribution to the total interaction energy originates from the frozen term, which is in stark contrast to the He@Au–PHI system. The polarization energy is also contributing to the total interaction energy, but is negligible (only  − 0.6 kJ mol^−1^) in He@H–PHI. The latter is most likely a consequence of its structure, where the H atoms are covalently bonded to the PHI framework. Most interesting, however, the polarization energy is significantly higher in both the He@Au–PHI and He@K–PHI systems, which can be ascribed to the strong polarizing environment due to the FCP structure. This clearly indicates that the strong polarized environment of M–PHI is indeed able to polarize the otherwise non-polarizable He atoms. However, based on our calculations, we find that the interactions between the He atoms and M–PHI system is purely non–bonding in nature and no formation of any chemical bond is observed.

**Figure 3:**
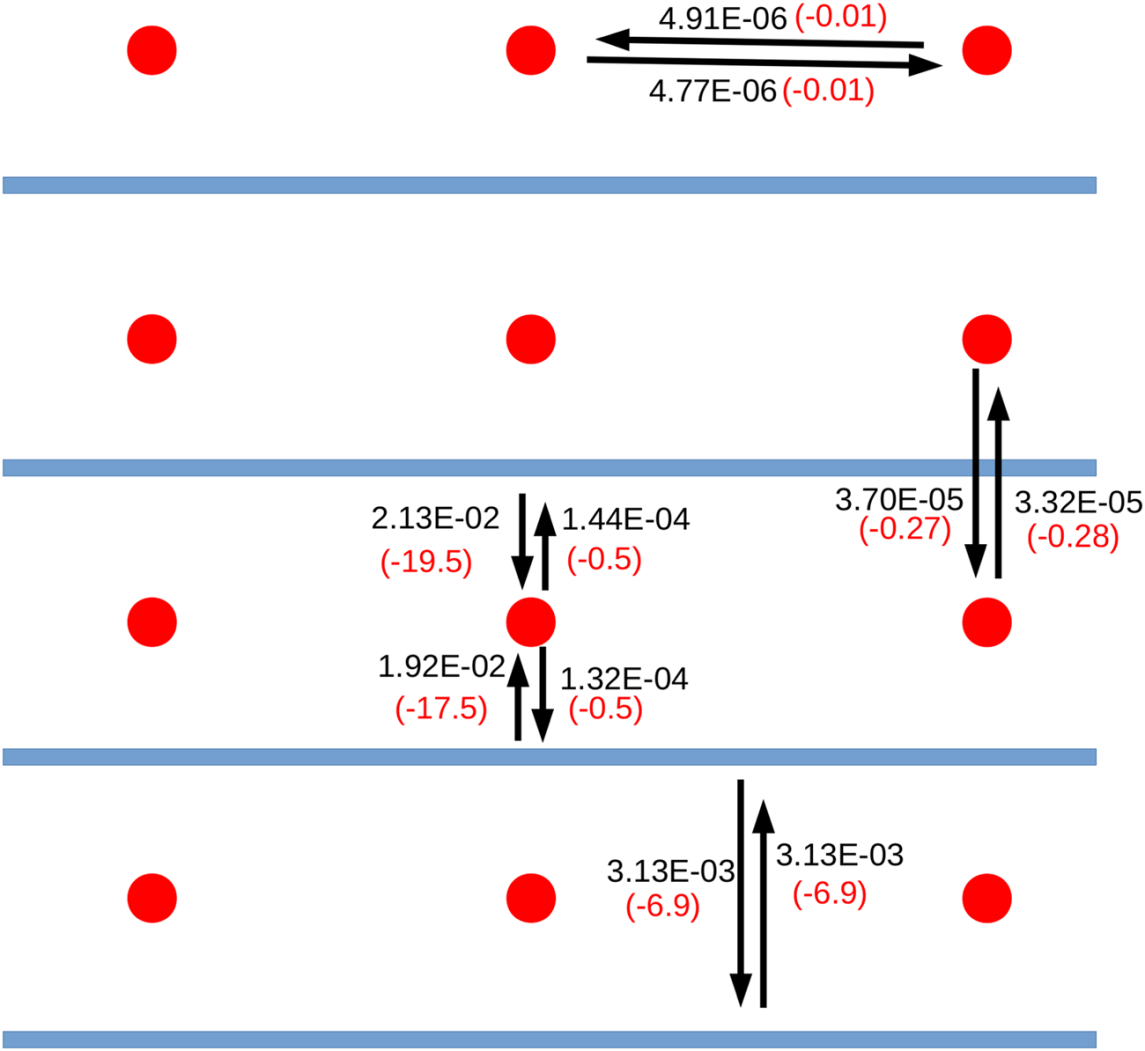
Schematic diagram showing the charge transfer (black color) in atomic units and the corresponding stabilization energy (red color) in kJ mol^−1^ between fragments of K–PHI, as computed using our ALMO–EDA method. The red spheres and the blue bars represent the K ions and PHI–layers, respectively. The arrows indicate charge transfer from the electron donor to the electron acceptor.

**Figure 4:**
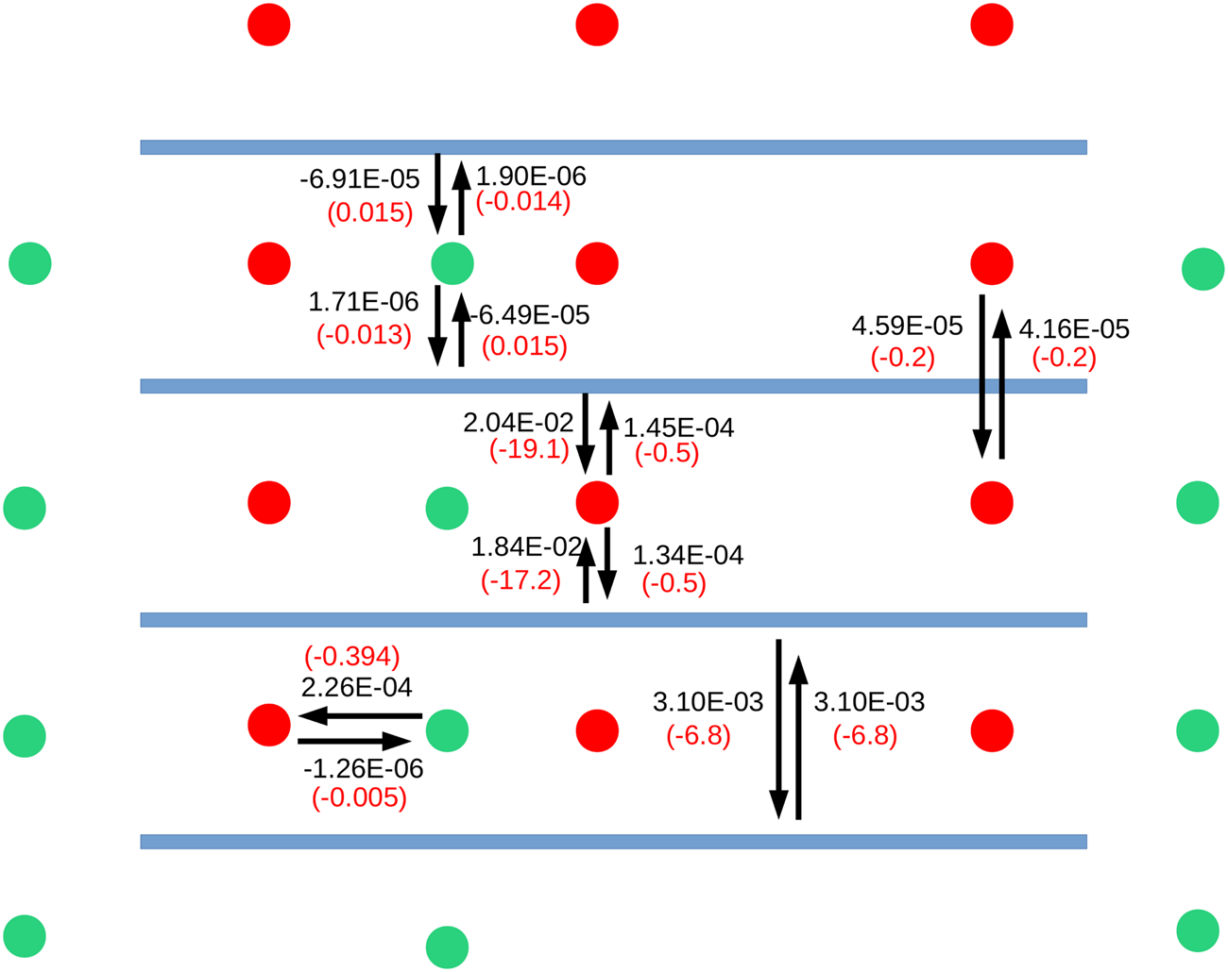
Schematic diagram showing the charge transfer (black color) in atomic units and the corresponding stabilization energy (red color) in kJ mol^−1^ between fragments of He@K–PHI, as computed using our ALMO–EDA method. The He atoms are denoted as green spheres.

The charge transfer contributions between the selected fragments of K–PHI and He@K–PHI and associated stabilization energies are schematically given in Figs. 3 and 4, respectively. The corresponding values for H–PHI and Au–PHI based control systems are reported in Supplementary Figs. S15–S18. First, we observe that there is sizable charge transfer between the PHI–layers, between the ions themselves and most interestingly between the embedded ions and the PHI–layers, i.e. the material in conductive. Our ALMO–EDA calculations unveil a small, but significant charge–transfer between the PHI–layers within all of the systems we have studied that varies from 3 me (K–PHI) to 5 me (H–PHI and Au–PHI). Yet, similar to liquid water^46^, even such seemingly small amount of charge transfer leads to an additional stabilization energy of 7 to 10 kJ mol^−1^. In all cases the interactions between the ions along the 2D PHI scaffold is throughout tiny and as such inessential. However, in the case of Au–PHI, some charge transfer between the Au ions perpendicular to the PHI plane of 9 me entailing a stabilization energy of 7.5 kJ mol^−1^ is detected. For K–PHI, these contributions between the K ions themselves are small and negligible. On the contrary, we observe a very large charge transfer of 21 me between the PHI–layers and the embedded K ions, which results in an extra stabilization energy of 20 kJ mol^−1^.

Interestingly and relevant for other catalytic experiments^47^, Au–PHI exhibits a huge charge transfer of 460 me, corresponding to an interaction energy of 247 kJ mol^−1^ that strongly binds the Au ions to the associated PHI–layer in a coordination complex-type fashion, but does only little to contribute to the stabilization of the whole structure. This binding also locates the gold ions (Au^+^) within the layers, rather than in between. It is therefore evident that the rather strong interaction within K–PHI is a direct consequence of its different ion distribution, also creating the FCP structure, where the K ions are located between the PHI–layers. This entails a symmetric and thus large stabilization of the 2D geometry. Moreover, this could also be the reasons why K ions can be replaced so easily by other ions in a fast ion-exchange process^14^. An important consequence of the above discussion is that by changing the ions from first and second main group to transition metals the interaction between the PHI–layers can be systematically tuned and thereby the layer distance and hence the band-gap changes^48^.

In He@K–PHI, i.e. when He atoms are adsorbed in K–PHI, they are at least partially confined between the PHI–layers by means of charge transfer contributions with the PHI scaffold (the “base” side) and more importantly with the K ions (the “acid” side). As can be seen in Fig. 4, the main contribution is due to the charge transfer between the He atoms and K ions, which amounts to 0.23 me corresponding to a stabilization energy of 0.4 kJ mol^−1^. Comparing this value for He@K–PHI with the one of He@Au–PHI, where the charge transfer is just 0.01 me, it becomes clear that it is the charge frustrated structure of He@K–PHI, where both the K ions and the He atoms are located between the PHI–layers, is essential for sizable adsorption effects and binding enthalpies. Interestingly, the He atoms are found to be much better electron donors than electron acceptors, which goes with the general chemical expectation.

In conclusion, based on an idealized structural model derived from powder X–ray diffraction, we found energetically optimized atomic structures of H–PHI, Au–PHI and K–PHI and investigated the possibility of these materials to adsorb He by means of theoretical first-principles calculations. We found that in H–PHI, the H atoms are covalently bonded to the N atoms of the PHI–scaffold, well confirming its weak acidic nature. In Au–PHI, the Au ions are present in the same plane as the 2D PHI–layers, which was attributed to some coordinative binding to the layer with significant electron transfer to the gold. Interestingly in K–PHI, the K ions are located between the PHI–layers, which is semi-quantitatively in good agreement with PXRD measurements. Population analysis shows that an unusually large amount of partial charge is moved from the ions to the electron–poor PHI scaffold, making the K^+^ ions even more positive and resulting both in improved charge transfer and higher thermodynamic stability. Due to the involved distance between the charges, the charge do not form contact pairs, but rather frustrated Coulomb pairs, very similar to the known and well described FLPs. In the present case, the PHI framework has clearly base character, while the excessivly positive K^+^ takes the role of a Lewis acid.

Based on ALMO–EDA calculations we were able to rigorously establish the existence of a rather high charge transfer induced binding between the PHI–layers and the embedded ions, as well as between the ions and the PHI scaffold. The second particularly important interaction, is most pronounced in K–PHI due to its FCP structure. In any case, all of these interactions contribute to stabilize all considered PHI–based systems beyond conventional aromatic stacking between the PHI–layers, and this is also reflected in the tighter interlayer packing of carbon nitrides, which is 5% shorter than in crystalline graphite, a big difference in the world of bond lengths. It has not escaped our attention that this immediately suggest the possibility to systematically tune these charge transfer induced interactions between the PHI–layers and thereby the layer distance and hence the band-gap.

Moreover, we also showed that all of the considered systems may be suitable candidate for He adsorption of up to 8 at%, though the adsorption energy is generally rather weak and only for K–PHI with  − 4.6 kJ mol^−1^ per He atom beyond *k*_B_*T* at ambient conditions. Interestingly, the adsorption of He within K–PHI is again governed by the intricate interplay of K–PHI’s FCP structure and sizable partial charge transfer between the He atoms and K ions, which is surprising in spite of the non-polar and usually non-polarizeable nature of He. We conclude by noting that He adsorption within PHI–based systems can potentially be further improved by replacing K with other cations that also adopts FCP structures, but are able to polarize He even more.

## Methods

### Computational details

Periodic DFT calculations were carried out using the hybrid Gaussian and plane wave approach^49^, as implemented in the CP2K/Quickstep code^50,51^. The Kohn-Sham orbitals were described by an accurate molecularly optimized double-zeta basis set with one additional set of polarization function^52^, while the charge density was represented by plane waves with a density cutoff of 500 Ry. The B97-D exchange and correlation functional, which is based on Becke’s power-series Ansatz, plus a damped atom-pairwise dispersion correction to account for long-range van der Waals interactions was employed^53^. Separable norm-conserving pseudopotentials were used to mimic the interactions between the valence electrons and the ionic cores^54^. The M–PHI (M = K, Au and H) structure was modeled using a supercell of *a* = *b* = 12.5 Å, *c* = 12.8 Å, *α* = *β* = 90.0° and *γ* = 120.0° which consists of 4 PHI–layers. Optimized structures were obtained by globally minimizing the potential energy, while varying the atomic positions by dynamical simulated annealing based on the second-generation Car-Parrinello approach of Kühne *et al*.^30,31,55^.

### Energy decomposition analysis based on absolutely localized molecular orbital

In order to investigate the nature of interactions in He@M–PHI, we have also conducted ALMO–EDA calculations for condensed phase systems^42^. In ALMO–EDA, the total interaction energy

$$\Delta {E}_{{\rm{TOT}}}=\Delta {E}_{{\rm{FRZ}}}+\Delta {E}_{{\rm{POL}}}+\Delta {E}_{{\rm{CT}}}$$

is decomposed into chemically meaningful components, such as the frozen interaction term *ΔE*_FRZ_, which is defined as the energy required to bring isolated molecules into the system without any relaxation of their molecular orbitals (apart from modifications associated with satisfying the Pauli exclusion principle), and an orbital relaxation contribution. The later quantity is then further decomposed into a polarization term *ΔE*_POL_, which is defined as the energy lowering due to the relaxation of each molecule’s ALMOs in the field of all other molecules, and the charge-transfer *ΔE*_CT_ contribution that is calculated as the difference in the energy of the relaxed ALMO state and the state of fully delocalized optimized orbitals.

A distinctive feature of the ALMO–EDA is that the charge-transfer contribution can be separated into terms associated with forward- and back-donation for each pair of molecules, as well as a many-body higher-order (induction) contribution *ΔE*_HO_, which is very small for typical intermolecular interactions. Both, the amount of electron density transferred between a pair of molecules *ΔQ*_CT_ and the corresponding energy lowering *ΔE*_CT_ can be computed via

$$\Delta {E}_{{\rm{CT}}}=\sum _{x.y > x}\left\{\Delta {E}_{x\to y}+\Delta {E}_{y\to x}\right\}+\Delta {E}_{{\rm{HO}}}$$

$$\Delta {Q}_{{\rm{CT}}}=\sum _{x.y > x}\left\{\Delta {Q}_{x\to y}+\Delta {Q}_{y\to x}\right\}+\Delta {Q}_{{\rm{HO}}}.$$

## Supplementary information

Supplementary Information.

## Data Availability

The datasets generated and analysed during the current study are available from the corresponding author on reasonable request.
