# Supplementary material for: On the Possibility of Helium Adsorption in Nitrogen Doped Graphitic Materials
Source: Sci Rep. 2020 Apr 2;10:5832. doi: 10.1038/s41598-020-62638-z (PMC7118168; doi:10.1038/s41598-020-62638-z)
Supplement: Supplementary file 1 — Supplementary Information. [file 41598_2020_62638_MOESM1_ESM.pdf]

Supplementary Information: On the Possibility of Helium Adsorption in Nitrogen Doped Graphitic Materials

Sudhir K. Sahoo,[†] Julian Heske,[†,‡] Sam Azadi,[¶,§] Zhenzhe Zhang,[‖] Nadezda V. Tarakina,[‡] Martin Oschatz,[‡,⊥] Rustam Z. Khaliullin,[‖] Markus Antonietti,[‡] and Thomas D. Kühne[*,†,#]

†*Dynamics of Condensed Matter and Center for Sustainable Systems Design, Chair of Theoretical Chemistry, University of Paderborn, Warburger Str. 100, D-33098 Paderborn, Germany*

‡*Department of Colloid Chemistry, Max Planck Institute of Colloids and Interfaces, Am Mühlenberg 1, D-14476 Potsdam, Germany*

¶*Department of Physics, King's College London, Strand, London WC2R 2L, United Kingdom*

§*Department of Physics, Imperial College London, Exhibition Road, London SW7 2AZ, United Kingdom*

‖*Department of Chemistry, McGill University, 801 Sherbrooke Str. West, Montreal, Quebec H3A 0B8, Canada*

⊥*University of Potsdam, Institute of Chemistry, Karl-Liebknecht-Str. 24–25, D-14476 Potsdam, Germany*

#*Paderborn Center for Parallel Computing and Institute for Lightweight Design, University of Paderborn, Warburger Str. 100, D-33098 Paderborn, Germany*

E-mail: tdkuehne@mail.upb.de

Structure of M–PHI

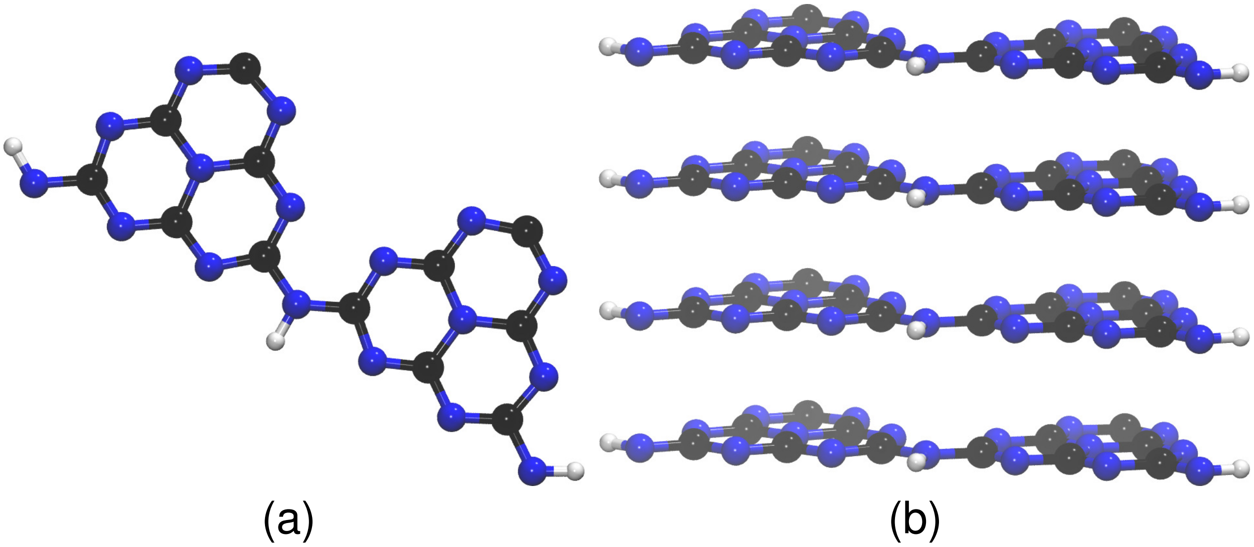

Figure S1: The bulk structure of H–PHI (top view(a) and side view(b)). Atoms color: C-black, N-blue and H-white.

Figure S3 shows the crystal structure of K–PHI, distribution of K ions, obtained from powder X-ray diffraction. It is noted that powder X-ray diffraction data shows that the Figure S2c is the most probable structure rather than Figure S2d.

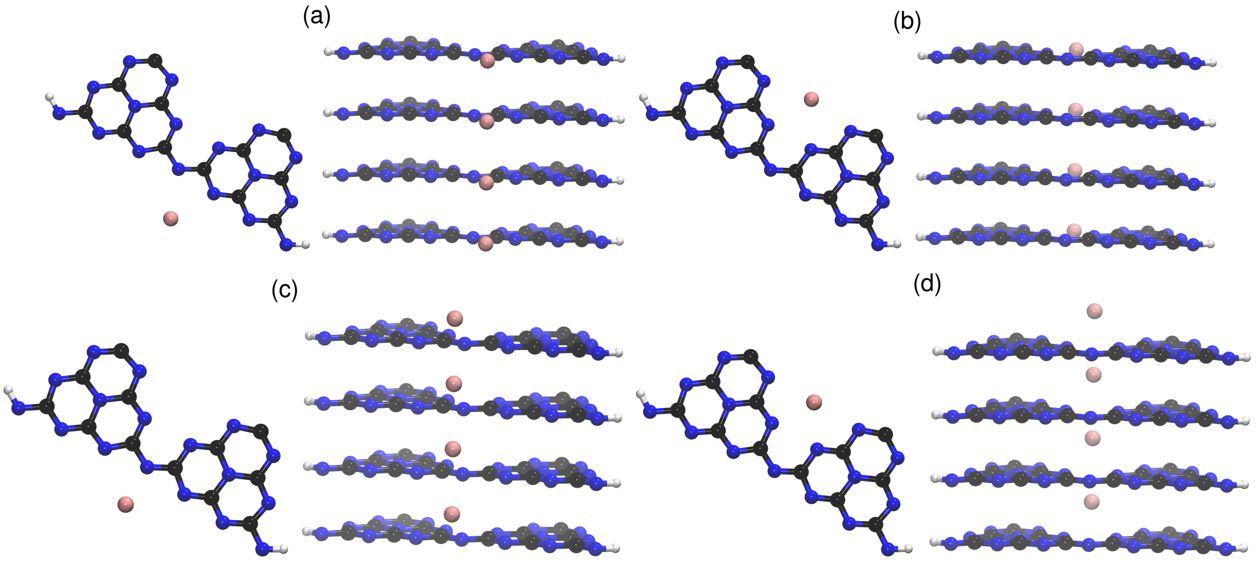

Figure S2: The optimized structure of K–PHI (one of the proton is replaced by one K ion in H–PHI) varying the position of K ion, the K ion is either present in the same plane of PHI; (a) 0.00 kJ $mol^{-1}$and (b) 0.10 kJ mol^{-1} or between the PHI-planes; (c) -169.49 kJ mol^{-1} and (d) -245.29 kJ mol^{-1}.

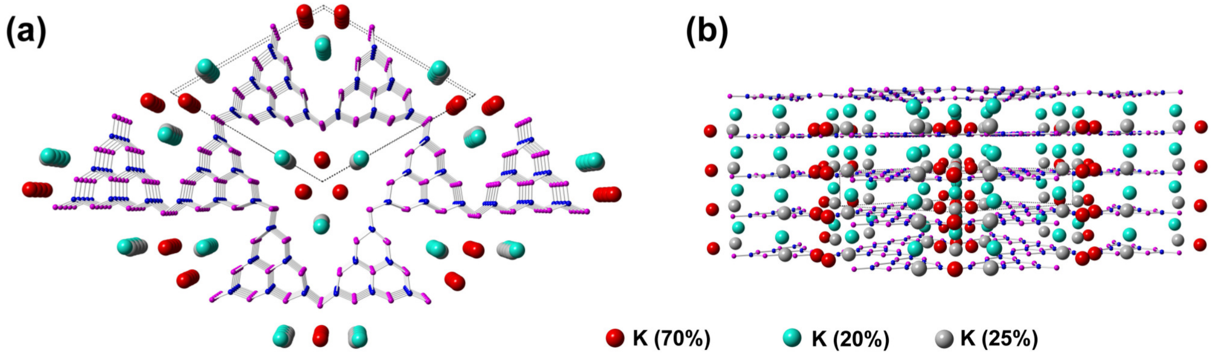

Figure S3: Crystal structure of K–PHI; view along (a) the [001] direction, (b) the [110] direction.

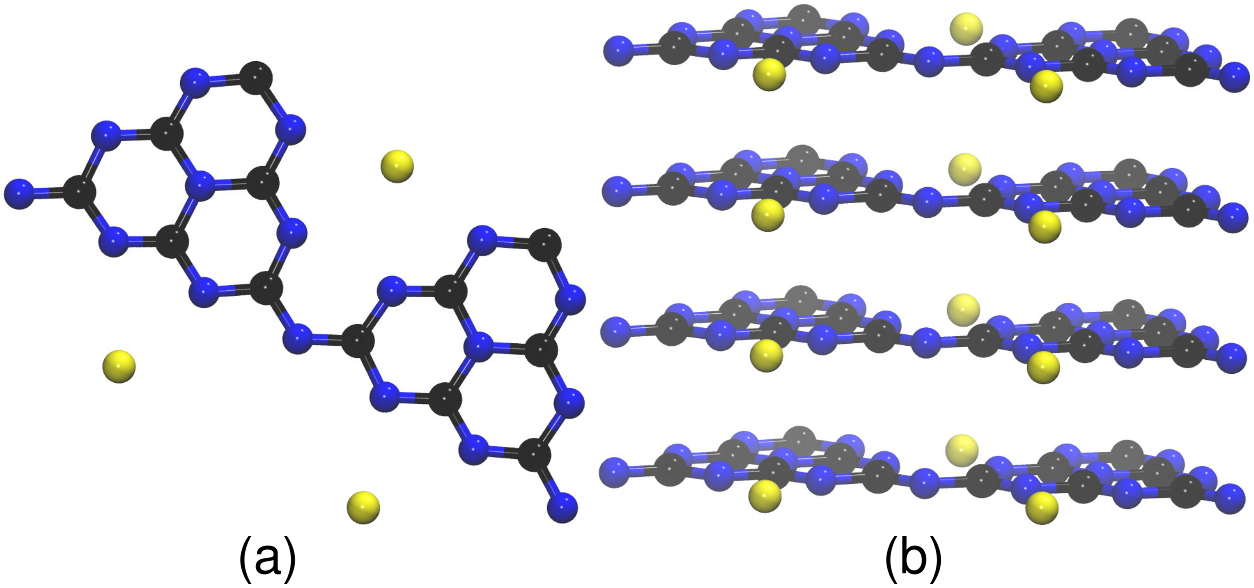

Figure S4: The bulk structure of Au–PHI, where all Au ions are present in the same plane of PHI (top view(a) and side view(b)). Atoms color: C-black, N-blue and Au-yellow.

Helium Adsorption in M-PHI

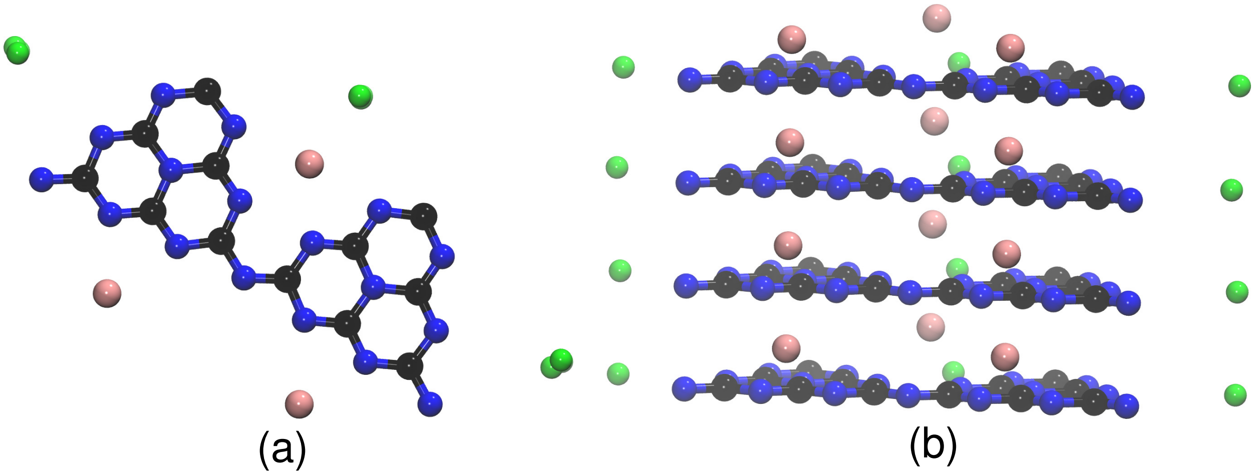

Figure S5: Structure of He@K–PHI ((a) top view and (b) side view), where all adsorbed He atoms present in the same plane of PHI.

Table S1: Adsorption energy in kJ mol^{-1} for He adsorption in H–PHI.

No. of He atoms	He atoms in the same PHI–plane		He atoms between the PHI–plane	
	ΔE_{tot}^{ads}	ΔE_{incr}^{ads}	ΔE_{tot}^{ads}	ΔE_{incr}^{ads}
1	-2.58	-2.58	-2.44	-2.45
2	-2.60	-2.63	-2.50	-2.56
3	-2.62	-2.66	-2.52	-2.56
4	-2.67	-2.82	-2.56	-2.68
5	-2.52	-1.93	-2.70	-3.26
6	-2.44	-2.01	-2.82	-3.40
7	-2.40	-2.13	-2.87	-3.19
8	-2.36	-2.13	-2.96	-3.57
9	-2.24	-1.28	-2.93	-2.73
10	-2.15	-1.38	-2.92	-2.80
11	-2.07	-1.21	-2.95	-3.28
12	-2.01	-1.41	-2.97	-3.11

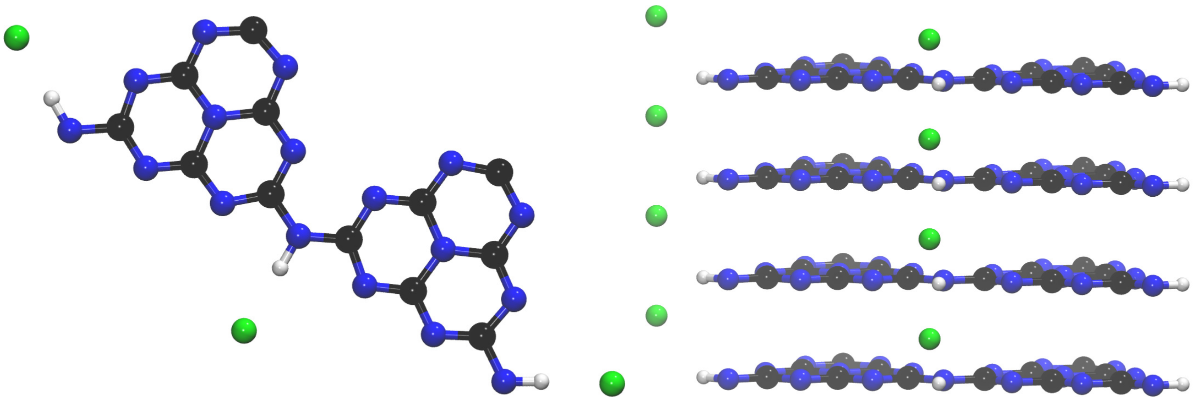

(a) (b)

Figure S6: Structure of He@H–PHI ((a) top view and (b) side view), where all adsorbed He atoms are present between the PHI planes.

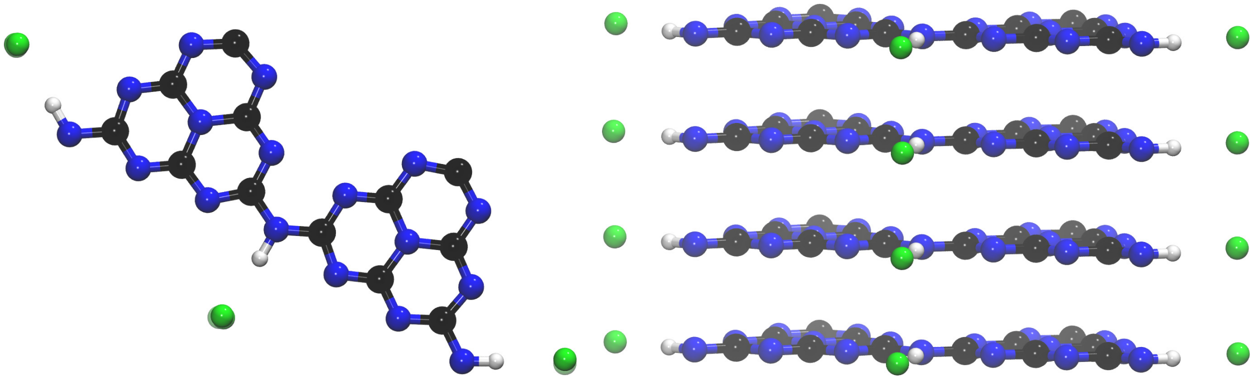

(a) (b)

Figure S7: Structure of He@H–PHI ((a) top view and (b) side view), where all adsorbed He atoms present in the same plane of PHI.

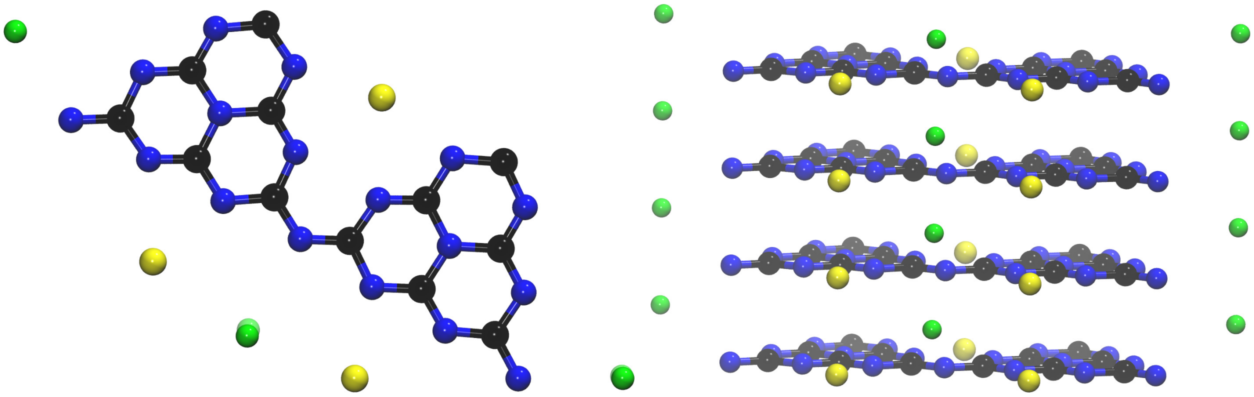

(a) (b)

Figure S8: Structure of He@Au–PHI (top view (a) and side view (b)), where 12 He atoms are adsorbed in Au–PHI, all the He atoms are present between PHI layers. Atoms color: C-black, N-blue, Au-yellow and He-green.

Table S2: Adsorption energy in kJ mol^{-1} for He adsorption in Au–PHI.

No. of He atoms	He atoms in the same PHI–plane	
	ΔE^{ads}_{tot}	ΔE^{ads}_{incr}
1	-3.09	-3.09
2	-3.08	-3.07
3	-3.09	-3.08
4	-3.09	-3.08
5	-2.63	-0.80
6	-2.29	-0.57
7	-2.04	-0.57
8	-1.86	-0.59
9	-1.77	-1.05
10	-1.69	-0.96
11	-1.63	-0.99
12	-1.61	-1.43

Quantum Monte Carlo Calculations

We solved the imaginary–time Schrodinger equation using the stochastic method of diffusion quantum Monte Carlo (DMC). The fixed-node approximation is applied to take into account the anti-symmetry property of many electronic wave function (WF). We used the Slater-Jastrow trial WF,

$$\Psi_{\mathrm{SJ}}(\mathbf{R}) = \exp[J(\mathbf{R})] \det[\psi_n(\mathbf{r}_i^{\uparrow})] \det[\psi_n(\mathbf{r}_i^{\downarrow})] \tag{1}$$

where $\mathbf{R}$ defines the positions of all electrons, $\mathbf{r}_i^{\uparrow}$ and $\mathbf{r}_j^{\downarrow}$ are the positions of the ith spin-up and $j$the spin-down electrons, respectively, $\exp[J(\mathbf{R})]$ is the Jastrow factor and $\det[\psi_n(\mathbf{r}_i^{\uparrow})]$ and $\det[\psi_n(\mathbf{r}_i^{\downarrow})]$ are Slater determinants of spin-up and spin-down single electron orbitals. The one-electron orbitals were obtained from DFT calculations using a norm-conserving pseudopotential with the PBE XC functional and a large basis-set cut-off of 300 Ryd to achieve the complete basis set limit.

The Jastrow factor was formed of polynomial one-body electron-nucleus and two-body electron-electron terms. The parameters of the Jastrow were optimised by variance minimization at the variational Monte Carlo (VMC) level.[1] The DMC calculations were carried out using the CASINO QMC package.[2] In all DMC calculations a time step of $\tau = 0.01$ Hartree atomic units of time was used.

References

(1) Umrigar, C. J.; Wilson, K. G.; Wilkins, J. W. *Phys. Rev. Lett.* **1988**, *60*, 1719–1722.

(2) Needs, R. J.; Towler, M. D.; Drummond, N. D.; Ríos, P. L. *J. Phys.: Condens. Matter* **2010**, *22*, 023201.

Mulliken Population Analysis

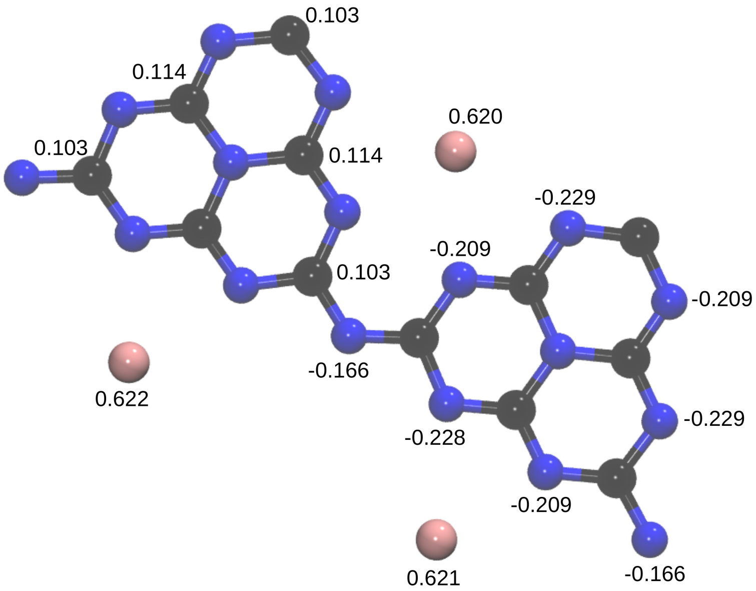

Figure S9: Mulliken population analysis of K–PHI.

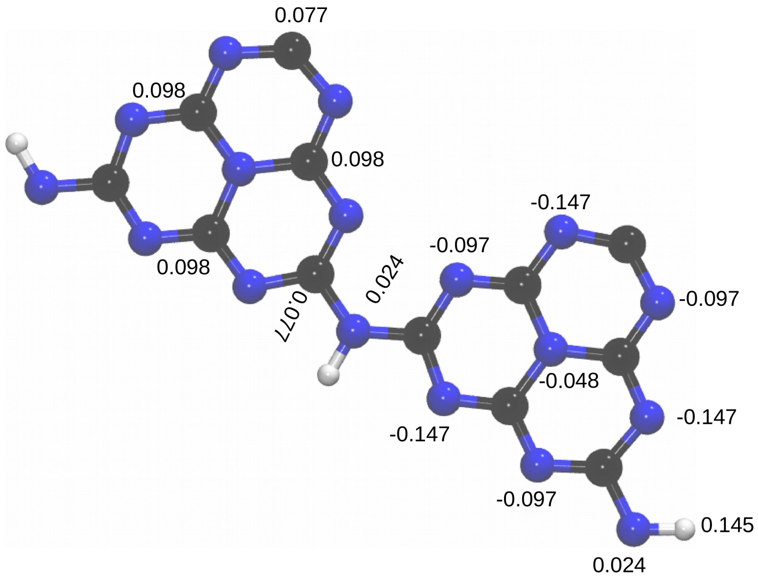

Figure S10: Mulliken population analysis of H–PHI.

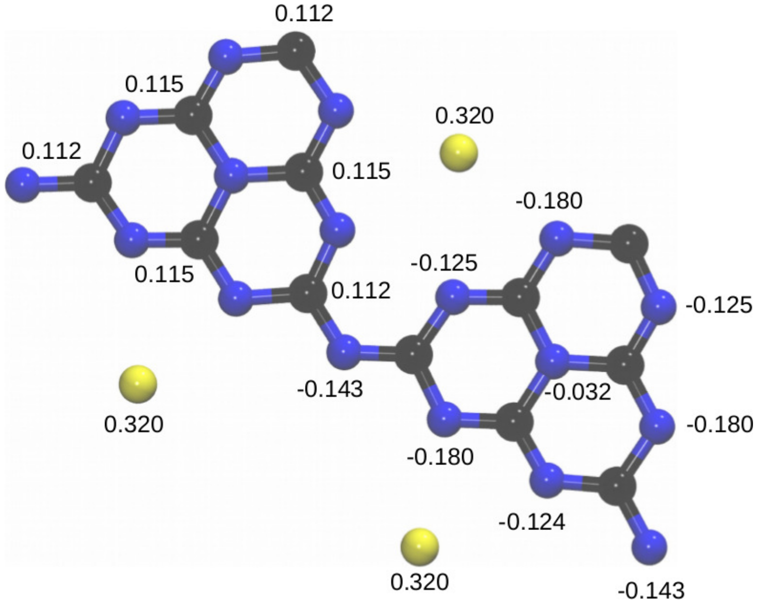

Figure S11: Mulliken population analysis of Au–PHI.

DDEC Charge Analysis

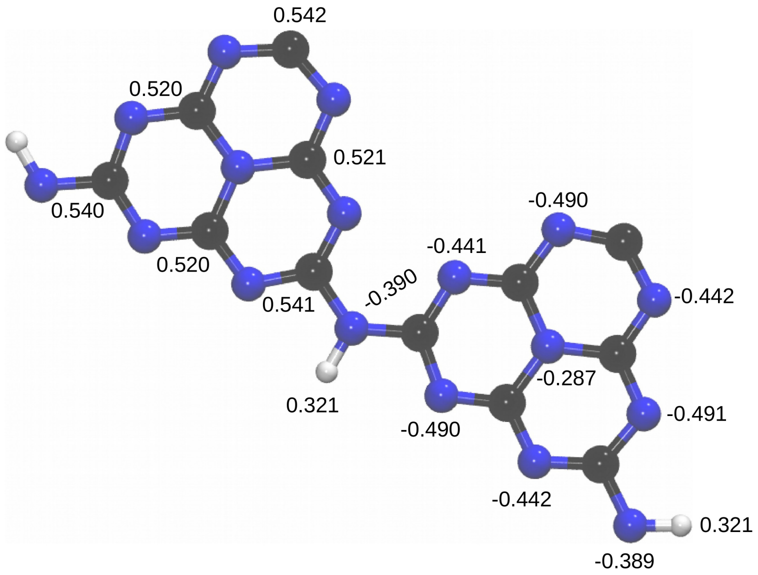

Figure S12: DDEC6 charge analysis of H–PHI.

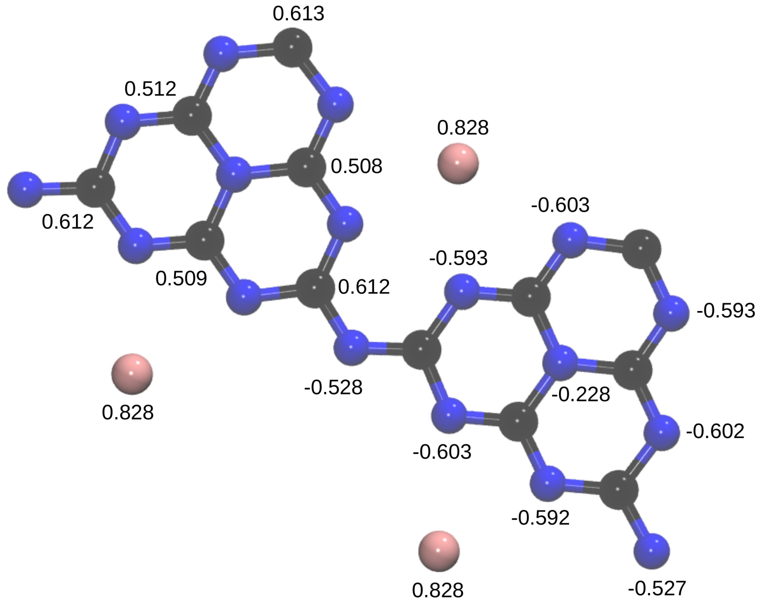

Figure S13: DDEC6 charge analysis of K–PHI.

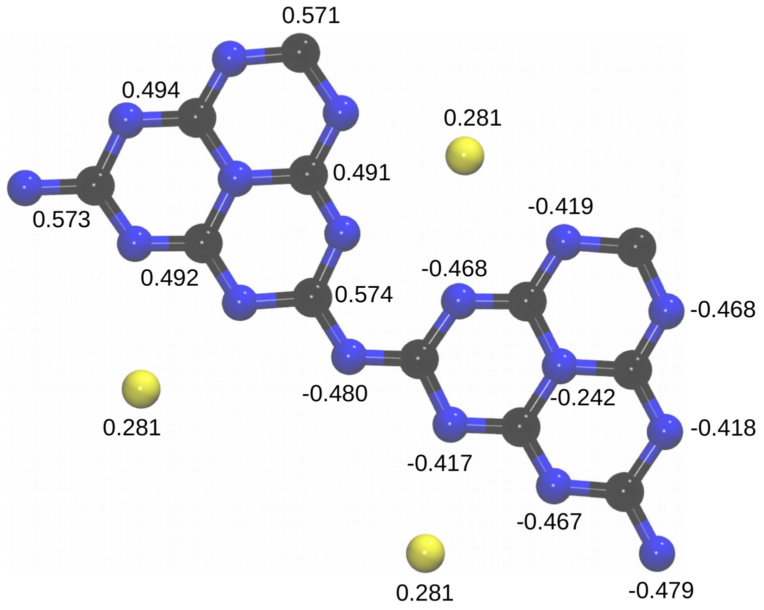

Figure S14: DDEC6 charge analysis of Au–PHI.

ALMO EDA Results

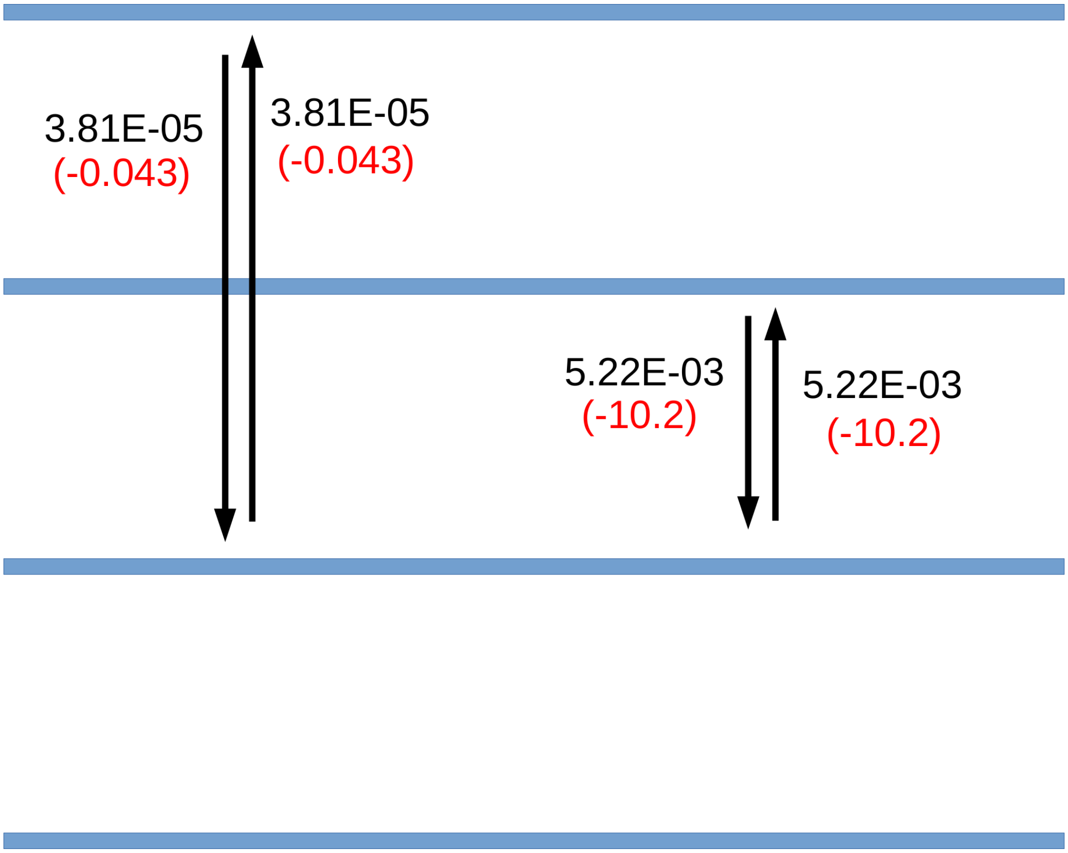

Figure S15: Schematic diagram showing the charge transfer (black color) in atomic units and the corresponding stabilization energy (red color) in kJ mol^{-1} between fragments of H–PHI, as computed using our ALMO–EDA method.

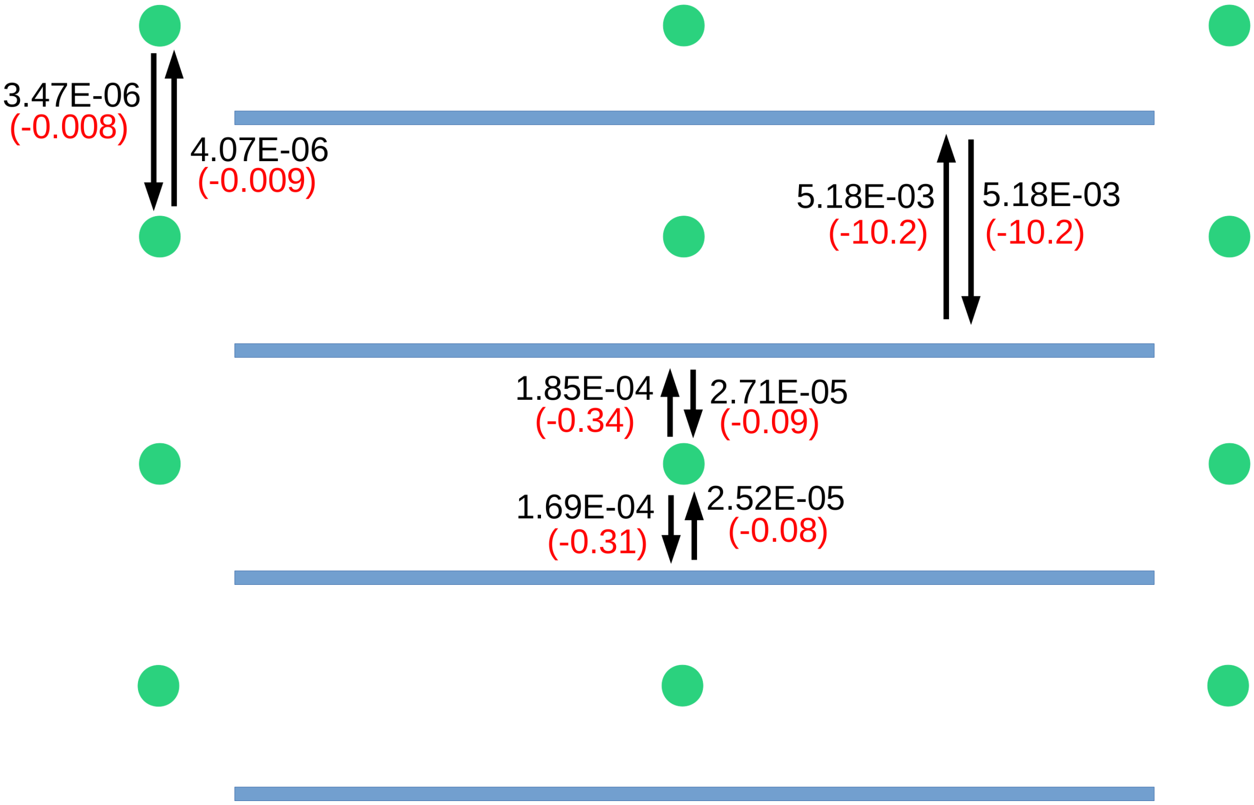

Figure S16: Schematic diagram showing the charge transfer (black color) in atomic units and the corresponding stabilization energy (red color) in kJ mol^{-1} between fragments of He@H–PHI, as computed using our ALMO–EDA method.

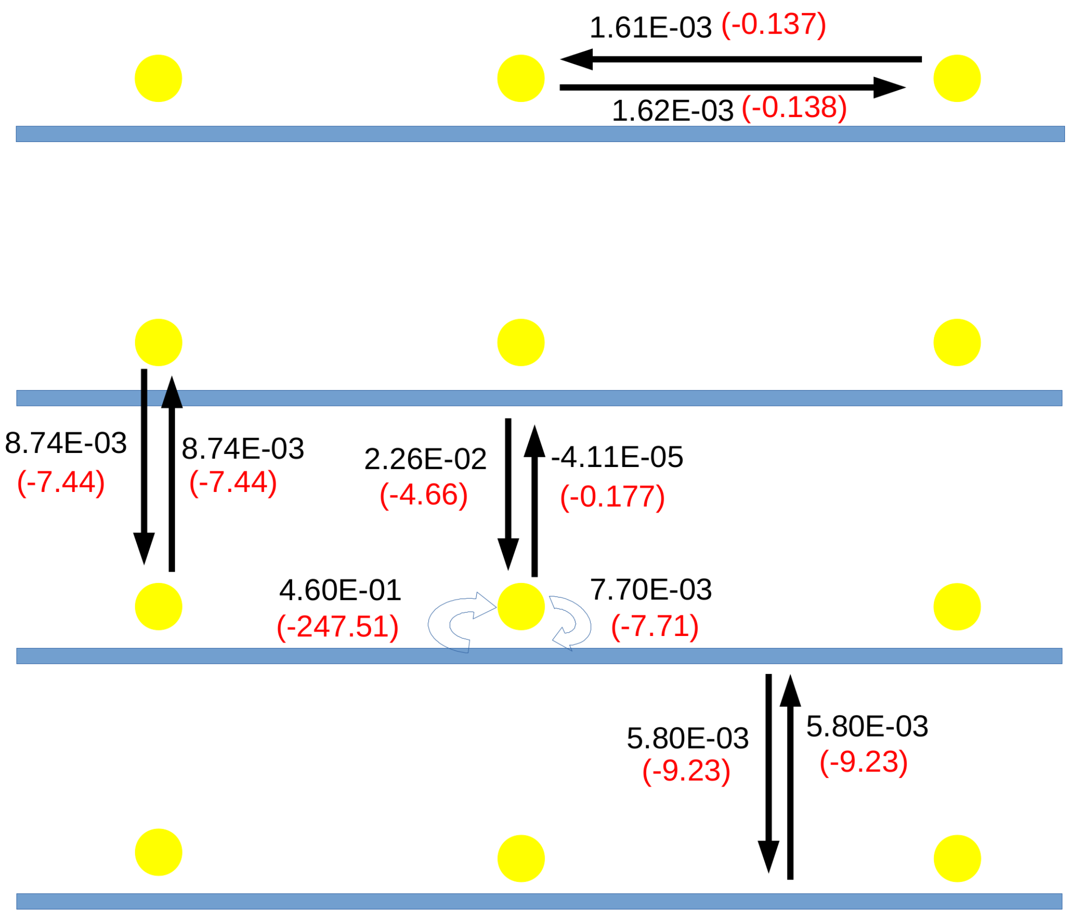

Figure S17: Schematic diagram showing the charge transfer (black color) in atomic units and the corresponding stabilization energy (red color) in kJ mol^{-1} between fragments of Au–PHI, as computed using our ALMO–EDA method. The yellow spheres represents the Au atoms.

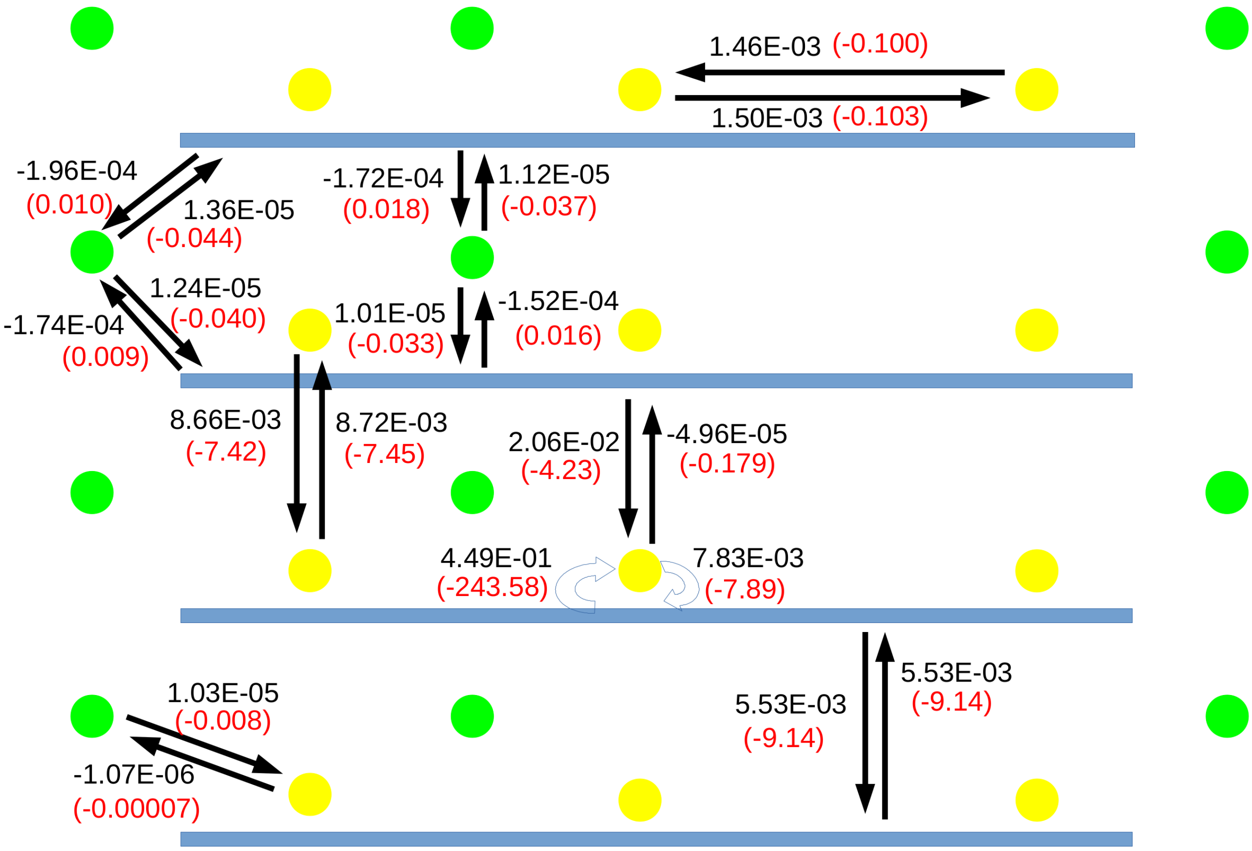

Figure S18: Schematic diagram showing the charge transfer (black color) in atomic units and the corresponding stabilization energy (red color) in kJ mol^{-1} between fragments of He@Au–PHI, as computed using our ALMO–EDA method.
